# Minimal Processing Technologies for Production and Preservation of Tailor-Made Foods^§^

**DOI:** 10.17113/ftb.61.03.23.8013

**Published:** 2023-09

**Authors:** Daniel Berdejo, Diego García-Gonzalo, Nadia Oulahal, Rositsa Denkova-Kostova, Vesela Shopska, Georgi Kostov, Pascal Degraeve, Rafael Pagan

**Affiliations:** 1 Departamento de Producción Animal y Ciencia de los Alimentos, Facultad de Veterinaria, Instituto Agroalimentario de Aragón-IA2, Universidad de Zaragoza-CITA (UNIZAR), C. de Pedro Cerbuna, 12, 50009 Zaragoza, Spain; 2 Université de Lyon, Université Claude Bernard Lyon 1 (UCBL), ISARA Lyon, BioDyMIA Research Unit, Technopole Alimentec, 155 rue Henri de Boissieu, 01000 Bourg en Bresse, France; 3 University of Food Technologies (UFT), 26 Maritza boulevard, Plovdiv, Bulgaria

**Keywords:** tailor-made food, biopreservation, emerging technologies, minimal processing technologies

## Abstract

Tailor-made foods, also known as foods with programmable properties, are specialised systems with unique composition prepared by different methods, using the known mechanisms of action of their bioactive ingredients. The development of tailor-made foods involves the evaluation of individual components, including bioactive substances derived from waste products of other productions, such as essential oils. These components are evaluated both individually and in combination within food compositions to achieve specific functionalities. This review focuses on the application of minimal processing technologies for the production and preservation of tailor-made foods. It examines a range of approaches, including traditional and emerging technologies, as well as novel ingredients such as biomolecules from various sources and microorganisms. These approaches are combined according to the principles of hurdle technology to achieve effective synergistic effects that enhance food safety and extend the shelf life of tailor-made foods, while maintaining their functional properties.

## INTRODUCTION

### Tailor-made foods – definition, development principles and technologies

By definition, a product can be called a tailor-made product if it is developed, adapted or suitable for a specific purpose or person. In the case of food development, it is most often assumed that tailor-made foods are foods developed for certain groups of consumers, which have been given certain functional characteristics (beyond their basic nutritional properties) and have been obtained/processed using appropriate technologies for maximum preservation of required functional/biological properties (*1*). To some extent, the term ‘tailor-made foods’ overlaps with the term ‘functional foods’. The term ‘functional foods’ was introduced in Japan for a special group of food products that were defined as follows: ‘food containing an ingredient with functions for health and officially approved to claim their physiological effects on the human body’. This terminology is also expressed in other documents in the USA and the European Union, but without the formalisation in the relevant documents (*1*, *2*). Currently, the term ‘functional’ is added to food products that have specific health benefits beyond the basic nutritional value of the product. Although there are currently legal definitions of a functional product (*1*, *3*-*7*), it is difficult to find a definition for the so-called tailor-made foods. In some cases, the concept of tailor-made foods is replaced by foods with programmable properties, although there is also no precise definition for the second concept. Therefore, it is possible to give the following definition: tailor-made foods (foods with programmable properties) are systems, the composition of which has been developed by various methods and means, by evaluating the activity of individual components (bioactive components, including the use of waste products from other productions, possessing a certain biological potential (*e.g*. essential oils), both individually and in the composition of foods, to achieve a certain functionality).

To obtain foods with programmable properties (tailor-made foods), it is necessary to follow four stages of development (*8*): (*i*) identification of the chemical, physical, nutritional, microbiological and functional properties and of the main ingredients in the target food and in the reference food product, (*ii*) development of databases of the main ingredients in the food and their influence on the functions of the food system, (*iii*) determination of appropriate combinations of basic components based on mathematical modelling and optimisation, and (*iv*) choosing a combination of components for the development of a nutritional system with a programmable property (functional foods).

**Fig. 1:**
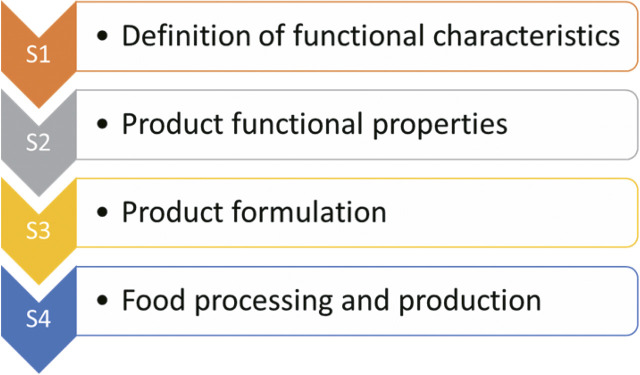
Basic steps for the production of tailor-made foods, adapted from Salmerón (*10*)

Akterian (*8*) and Avramenko and Kraslawski (*9*) suggested that the development of a new product (tailor-made food) includes 4 stages (Fig. 1 (*10*)).

In methodological terms, tailor-made foods can be obtained by targeted fermentation, adding functional ingredients in various forms to the composition of the food matrix. Increasing the biological activity of certain components or its preservation in the food production process or the fermentation capacity of lactic acid bacteria and yeast without genetic changes can be achieved by encapsulation technologies. From a methodological point of view, the preparation of this type of active ingredients can be achieved by the following approaches: examining suitable carriers for inclusion of microorganisms, bioactive substances, fats (*e.g.* containing polyunsaturated fatty acids) and flavouring substances; study of the stability of the resulting encapsulated systems as well as the metabolic changes resulting from the encapsulation of microorganisms (*10*-*16*).

From the point of view of the technological operations used, they do not differ significantly from technological operations used in the production of standard food products. The maximum preservation of the intended functional properties is of great importance for the production of tailor-made foods. In this sense, their production is related to the application of concepts and developing technologies aimed at precisely this. Biopreservation using microorganisms and/or biomolecules (of plant, animal or microbial origin), the use of hurdle technologies to preserve the final food product, use of emerging non-thermal technologies (*e.g*. high hydrostatic pressure, irradiation and others) to preserve the biological properties of the final product can be enlisted as examples of this concept (*17*-*20*).

The focus of the present work is on the development of tailor-made foods through the application of a knowledge system in the field of biopreservation, hurdle technologies and minimal food processing (*21*, *22*).

The aim of this work is to consider minimal processing approaches for the production and preservation of tailor-made foods and to describe the synergistic effect of different processes.

## HURDLE TECHNOLOGY PRINCIPLES AND THEIR POTENTIAL FOR PRESERVATION OF PERISHABLE/TAILOR-MADE FOOD

The concept of hurdle technology has been known for a long time, but it allows for integrated food preparation and preservation. Food with programmable composition, stable and safe properties can be developed by applying this technology. In recent years, this integrated approach has found increasing application due to consumer demands for minimal food processing (*18*). Therefore, in this publication we will consider the possibilities of combining obstacles in the preservation process, which are also important in the production of tailor-made foods.

### Biological hurdles

Biopreservation with the help of natural or controlled microbiota, as well as different types of antimicrobial agents, is one of the techniques that, in addition to a preservative effect on foods, also has an impact on their functional properties (*18*). Biological hurdle can be one of two types – biological hurdle by microorganisms and biological hurdle by extracts from various raw materials or biomolecules. Microbial fermentation of foods by spontaneous fermentation or selected cultures makes it possible both to introduce a biological hurdle to the growth of undesirable microorganisms and to obtain a food product with beneficial properties for human health (*18*, *22*-*24*).

Improving the quality of food products can be related to the replacement of traditional preservation methods (including chemical substances used as preservatives) with natural alternatives (*24*). In a broader sense, biopreservation of food products is a method of preservation that uses the inhibitory activity of different types of biomolecules (of plant, animal, or microbial origin), the use of targeted fermentation with appropriate microorganisms, or a combination of these two approaches to inhibit the growth of pathogenic microorganisms (*18*).

In this part of the article, we will discuss the different biological hurdles that can be used in the production of tailor-made foods.

#### Antimicrobial extracts and biomolecules

The use of natural compounds for their potential application as natural food biopreservatives is gaining significant interest due to negative consumer attitudes towards some synthetic antimicrobials currently used as food preservatives. These biopreservatives are natural ingredients or additives that can both retain a broad spectrum of antioxidant and antimicrobial activities and have the ability to improve the quality and shelf life of perishable food (*25*).

A perfect natural antimicrobial agent should meet the following requirements: (1) be effective in its natural form at low concentrations, (2) be non-toxic, (3) be economical, (4) not cause sensory changes in the product, and (5) inhibit a wide range of pathogenic and spoilage microorganisms. A number of compounds from natural sources have demonstrated the potential to be applied for food preservation due to their proven antimicrobial properties against a broad range of foodborne pathogenic and spoilage microorganisms (*25*).

The antimicrobial extracts/biomolecules can be of different origins, such as microbial origin (isolated from bacteria, actinomycetes and fungi), plant origin or animal origin, and can ensure food safety because of their antimicrobial activity against a wide spectrum of foodborne pathogens and spoilage microorganisms (*26*). Biopreservatives of plant or animal origin, natural or controlled microﬂora, such as lactic acid bacteria and the corresponding antibacterial metabolites produced by them (lactic acid, bacteriocins) have proven ability to inhibit colour loss, delay lipid oxidation, prolong storage life and, ultimately, ensure food safety. It is very important that the newly identiﬁed natural bioactive compounds not only have an antimicrobial effect specifically against foodborne pathogenic microorganisms and spoilage agents, but also do not negatively affect the consumer's own gastrointestinal microflora (*26*, *27*).

Some natural antimicrobials have been reported to inactivate microorganisms without negatively affecting the organoleptic or nutritional properties of food (*28*). Nevertheless, in complex food matrices, active biocompounds can bind due to electrostatic or hydrophobic interactions to other food components like proteins or lipids. Unfortunately, this restricts the availability of many natural antimicrobials. Moreover, the use of various processing methods can lead to a reduction in antimicrobial activity. Nanodelivery systems have thus been developed to improve the activity of some natural antimicrobial molecules (*e.g.* essential oil nanoemulsions). In a number of cases, incorporation of nanoparticles has been shown to be an effective and safe antimicrobial delivery system (*26*, *27*).

Some phenolic compounds of plant origin alter the microbial cell permeability, thus causing the loss of biomolecules from the inside of the cells (for example ribose and sodium glutamate). Some plant phenolics interact with membrane proteins and induce their structure and function alteration, thus interfering with cell membrane functionality. Impaired membrane functions are related to disruptions in electron transport, nutrient uptake, synthesis of proteins and nucleic acids and enzyme activity (*27*, *29*).

Lipids can inactivate microorganisms by the following three mechanisms: disruption of the bacterial cell membrane, hindering DNA replication or inhibition of other intracellular targets (*27*).

Antimicrobial peptides interact with the microbial cell membrane by adopting amphipathic structures. Membrane rupture in some locations causes the leaching out of essential cell biocomponents (*27*, *30*). For example, antimicrobial activity of bacteriocin results from the generation of pores in the cytoplasmic membrane of the target microorganism. This, in turn, leads to the loss of low-molecular-mass intracellular components and ions, and the disruption of the proton motive force (*27*, *31*).

Natural antimicrobials of plant origin. Phenolics (ﬂavonoids and non-ﬂavonoids), terpenes, aliphatic alcohols, aldehydes, ketones, organic acids, saponins, thiosulﬁnates and glucosinolates are the main compounds that impart plant antimicrobial properties (*28*, *32*). Plants, their corresponding essential oils, by-products and secondary metabolites can delay or inhibit the growth of bacteria, yeast and moulds (*27*, *28*, *32*). Plant extracts, including essential oils, have been observed to inhibit Gram-positive bacteria to a greater extent than Gram-negative bacteria (*26*). What makes a molecule even more effective for biopreservation of food is the presence of both antioxidant and antimicrobial components in the same molecule (*29*). The antimicrobial properties of products of plant origin are generally accompanied by an increased antioxidant capacity (*27*, *33*).

Natural antimicrobial agents of animal origin. Most animal-derived antimicrobial systems have evolved as part of the natural host defense mechanisms. The list of animal-derived antimicrobials includes antimicrobial proteins and peptides (like lactoferrin, pleurocidin, defensins and protamine) that are considered a viable alternative to antibiotic resistance (*34*). Moreover, some of these compounds could quickly destroy the cellular lipid bilayer membranes even of fast-growing microorganisms (*28*). Some of them also exhibit antifungal and antiviral activities in addition to their strong antibacterial effect against both Gram-positive and Gram-negative bacteria (*27*, *35*).

Some enzymes have also demonstrated promising antimicrobial properties. For example, lactoperoxidase has been reported to have powerful antimicrobial activity against bacteria, fungi and viruses (*36*), and lysozyme is effective against food spoilage Gram-positive bacteria (*28*, *37*), Gram-positive bacteria being the main target for the lysozyme, while Gram-negative bacteria are resistant to lysozyme because of the lipopolysaccharide layer of their outer membrane that actually constitutes a physical barrier. It has been reported that the presence of surfactants and chelating agents such as EDTA (ethylenediaminetetraacetic acid) enhance the sensitivity of Gram-negative bacteria to lysozyme because these compounds can initiate membrane disruption (*26*, *27*).

Some animal-derived polysaccharides and lipids have also shown antimicrobial effects. Chitosan has polycationic structure and is applied as effective antifungal (*38*) and antibacterial agent (*39*). Food lipids can also inhibit the proliferation of microorganisms and thus prevent the growth of pathogens and spoilage microorganisms (*27*, *40*).

#### Natural preservatives of microbial origin

Natural preservatives produced by bacteria. Cationic bacteriocin interacts with anionic lipids in the membrane of Gram-positive bacteria and thus induces the formation of pores across cellular membranes (*27*). Nisin is produced by *Lactoccocus lactis* and acts against Gram-positive and spore-forming bacteria. Pediocins are synthesised by *Pediococcus* strains (*P. acidilactici* and *P. pentosaceus*) and have demonstrated significant efﬁciency against spoilage and pathogenic microorganisms, such as *Listeria monocytogenes, Enterococcus faecalis, Sttaphylococcus aureus* and *Clostridium perfringens* (*27*, *28*). Reuterin (3-hydroxypropionaldehyde), produced and secreted by *Lactobacillus reuteri*, shows a wide spectrum of antimicrobial activity against foodborne pathogenic and spoilage microorganisms. The antibacterial activity of lactic acid and other organic acids produced by starter culture bacteria, such as acetic and propionic acids, is due to their ability to cross cell membranes (at pH values below their pKa), resulting in reduced intracellular pH and disruption of the transmembrane proton motive force (*27*).

Natural preservatives produced by streptomycetes. Actinomycetes have the ability to synthesise a range of antifungal, antiviral, antitumour, anti-inﬂammatory, antioxidant, immunosuppressive, plant-growth-promoting and herbicidal compounds (*41*). *Streptomyces* sp. synthesises a broad range of bioactive metabolites, for example, the antifungal natamycin is produced by fermentation using *Streptomyces* species. Natamycin has been shown to be effective against almost all yeasts and moulds in food but is inactive against bacteria and viruses (*41*). Its use as a food preservative is authorised in the EU.

Natural preservatives from algae and fungi. Many derivatives of marine algae are indeed promising new antimicrobial agents with multiple applications, including the pharmaceutical and food industries (*27*). Similarly to fruits, vegetables, herbs and spices, the antimicrobial activity of algae is combined with the antioxidant potential of their compounds (presence of α-tocopherol, carotenoids, polyphenols, phycobiliproteins and vitamin C) (*27*).

Bacteriophages. Another new and promising category of food biopreservatives are bacteriophages or their antibacterial enzymes (phage lysins or enzybiotics), which can be used directly as antibacterial agents due to their ability to act on and destroy the bacterial membranes. Bacteriophages have great potential as natural biopreservatives and can be used in various food systems due to their ability to selectively control the growth of bacterial populations. They are a promising alternative to antimicrobials, mainly because they have an almost unique host range that gives them great specificity. Moreover, their use does not have negative impact on the environment as in the case of antibiotics. The application of both phages and their enzymes could reduce the use of antibiotics, which is especially desirable having in mind the alarming increase in resistance to antibiotics used in human medicine, veterinary medicine, agriculture and all processes of manufacturing, preservation and distribution of food (*42*, *43*).

Bacterial quorum-sensing inhibitors and antagonists. It is well-known that quorum sensing plays a major role not only in food spoilage and food-related pathogenesis, but also in biofilm formation, virulence regulation, antimicrobial peptide synthesis, genetic competence and transfer of conjugative plasmids, sporulation and symbiosis (*25*). Bacterial quorum-sensing signalling systems can be a powerful weapon in controlling the growth of undesirable food-related bacteria. Quorum-sensing inhibitors/antagonists are being developed for use as new food preservatives to maintain food integrity, extend shelf life and enhance food safety because quorum-sensing signalling molecules are ubiquitous in many known bacterial species and have been linked to food spoilage. There are two types of quorum-sensing inhibitors: natural and synthetic quorum-sensing inhibitors. The natural quorum-sensing inhibitors have been obtained from prokaryotic, animal, plant, marine organisms and fungi. Nevertheless, the toxicological status of quorum-sensing inhibitors should be examined. It is a prerequisite to their use in food preservation (*25*).

Application of antimicrobials in successful biopreservation strategies. The antimicrobial activity of a particular compound depends largely on its physicochemical properties such as polarity, hydrophilicity/hydrophobicity, volatility and acid-dissociation property. It is quite challenging to generalise about methods for antimicrobial application because of the varying properties and antimicrobial action spectra among naturally occurring antimicrobials, the difference in food composition and microstructure (*e.g.* fat and protein content, pH, water activity and homogeneity), process factors and storage conditions (*25*).

The perfect antimicrobial agent should meet a number of requirements: (*i*) it must be effective against the microbial target, (*ii*) it must function in the chosen food system, (*iii*) it must have no or minimal interactions with food components, (*iv*) it must be stable during food processing and finally (*v*) it must not contribute to the development of resistance in target microorganisms or representatives of the microflora (*25*).

#### Bioprotective cultures

Lactic acid bacteria of the genera *Lactobacillus*, *Streptococcus*, *Enterococcus* and *Lactococcus* are of fundamental importance for the production of fermented foods. Bifidobacteria, some *Escherichia coli* strains, representatives of the *Bacillus* sp., moulds (primarily *Aspergillus* sp.) and yeasts (*Saccharomyces* sp.) can also be used as bioprotective cultures (*25*, *44*-*48*). Mainly because of their metabolism (and its products such as organic acids, mainly lactic and acetic acids, bacteriocins, hydrogen peroxide, diacetyl, reuterin and other metabolites), representatives of the genera *Lactobacillus* and *Bifidobacterium* have become established as bioprotective cultures for food production (*17*, *25*, *48*, *49*). Microorganisms of these genera have GRAS status because they are part of the natural microbiota of a number of foods (*44*).

An important element in the development of tailor-made foods is the possibility of combining several effects of a given food component. Many strains of lactic acid bacteria (LAB) have been classified as probiotics and fermented foods are an excellent source of such LAB (*50*).

The metabolites of lactic acid bacteria have a preservative effect *via* different mechanisms (*17*, *51*). The produced organic acids (lactic and acetic acids) reduce the pH of the medium and create a selective barrier that prevents the growth of certain microorganisms. Lactic acid has an antimicrobial effect by disrupting the cytoplasmic membrane (*17*, *52*). The CO_2_ produced by the heterofermentative lactic acid bacteria creates an anaerobic atmosphere that does not allow the growth of aerobic species. The diacetyl produced by lactic acid bacteria has antimicrobial effect against *Listeria* sp., *Salmonella* sp., *Escherichia coli*, *Yersinia* sp. and *Aeromonas* sp., H_2_O_2_ has an antimicrobial effect manifested by oxidative stress on the cell membrane (*17*).

Bacteriocins are of scientific and practical importance for biopreservation of food products. They are metabolites produced by lactic acid bacteria that are capable of inhibiting the growth of closely related microorganisms (*17*, *51*). These metabolites have GRAS status and do not affect the sensory characteristics of food. They are active against a wide range of food spoilage microorganisms and food pathogens (*45*, *53*). The antimicrobial activity of bacteriocins is mainly due to electrostatic interactions with negatively charged phosphates of phospholipids that create pores in the cell membrane, leading to cell lysis (*17*). Bacteriocins are of practical importance in the biopreservation of cheese, yoghurt, some types of meat products (*54*-*56*) and nisin, which is approved as food preservative in many countries (*54*), has perhaps gained the greatest practical importance. Some enterococci also produce bacteriocins (*54*). In their work, Settanni and Corsetti (*57*) showed that bacteriocins are used for biopreservation (introduced as protective or starter cultures) in fermented and non-fermented vegetables, meat, sourdough, juices, canned products, *etc*.

Yeasts are the second major group of bioprotective microorganisms. Due to their specificity and ability to grow under stressful conditions, they are used as bioprotective cultures in food production. Different yeasts are antagonistic to different spoilage and pathogenic microorganisms. Their antagonistic effect is to the result of competition in the assimilation of substrates and the production of a wide class of antimicrobial components (*58*). In addition, yeast antagonism is also a result of pH changes caused by the production of organic acids, tolerance to high ethanol concentrations and the production of the so-called killer toxins (*58*-*63*). In their work, Muccilli and Restuccia (*58*) showed the application of a number of yeast species (*Candida pyralidae, Candida zeylanoides, Candida famata, Debaromyces hansenii, Debaromyces maramus, Hyphopichi. burtonii, Filobasidium floriforme, Kluyveromyces wickerhamii* (Kwkt), *Pichia membranifaciens, Saccharomyces cerevisiae* and others) as bioprotective cultures in the production of beverages, dairy products, olives, bread and bakery products (*58*). In most cases, the bioprotective effect is due to a combination of the already described mechanisms.

### Physical hurdles

#### Existing methods

Heat treatment is one of the most widely used physical methods for food preservation because it can effectively inactivate enzymes and microorganisms. Sterilisation, which requires high temperatures, is in fact currently one of the few technologies that can ensure the destruction of bacterial spores in food (*64*).

While the effectiveness of heat on inactivation of enzymes depends mainly on their thermostability, the inactivation of microorganisms can be more complex. Thus, the mechanisms of microbial inactivation by heat have been extensively studied and many different cellular alterations have been reported. It is generally assumed that heat disrupts multiple structures and functions of the microorganisms, with the sum of all changes leading to cell death. In general, when studying the effect of heat, the cellular structures or elements that have often been studied as susceptible are the outer and inner membrane, the peptidoglycan cell wall, nucleic acids (nucleoid and RNA), ribosomes and various enzymes (*65*).

Some of the limitations of conventional thermal treatments are uneven heating and low heat transfer efficiency in some foods, so more rigorous treatments must be applied to ensure that all parts of the whole food receive the minimum treatment required. In this regard, electromagnetic technologies have gained increased interest and demonstrated their potential for faster and more uniform heat treatments of food. Technologies such as infrared heating, microwave heating, radiofrequency heating and instant pressure drop control technology have shown to reduce these drawbacks and provide, at least in part, an alternative to conventional heat treatment in food preservation (*66*).

Despite advances in the use of thermal treatments, undesirable side effects of heat on the sensory, nutritional and functional properties of food could still occur (*67*). These changes in foods and the growing consumer demand for minimally processed foods have prompted the development of alternative, non-thermal preservation methods that offer safe foods with minimal processing treatments due to their antimicrobial efficacy (*68*). Technologies such as high hydrostatic pressure (HHP), pulsed electric field (PEF), ionising radiation (IR) and non-thermal atmospheric plasma (NTAP) have shown to be promising alternatives for food preservation.

#### Emerging technologies

High hydrostatic pressure. Industrial HHP processing is based on treatments with increased pressure (100–600 MPa) transmitted throughout the product using a pressure-transmitting medium with a duration of 1 to 20 min. These treatments at refrigeration or room temperature (4-25 °C) allow the extension of shelf-life and reduce microbial loads to levels comparable to those achieved by thermal pasteurisation, without affecting nutritional and sensory attributes (*69*). HHP treatments eliminate or inactivate vegetative forms of spoilage and pathogenic microorganisms, but not their spore form. However, HHP treatments have been shown to induce the germination of bacterial spores of several bacterial genera, such as *Bacillus* spp., and thus facilitate their inactivation by subsequent treatments (*70*).

The mechanisms of microbial inactivation by HHP are very complex and diverse. Microbial death is thought to be caused by the accumulation of all the damage to different cell structures and functions: disruption of the outer membrane, damage to the cytoplasmic membrane, alteration of pH homeostasis and osmoregulation, damage to cytoplasmic components (such as disintegration of ribosomes into their subunits, protein and enzyme unfolding) and damage to DNA and RNA by reactive oxidative stress (ROS) (*71*, *72*).

Nowadays, HHP is the most-widely used non-thermal technology for the commercial pasteurisation of food. According to Khouryieh (*73*), 35.6 % of food processing companies in the USA have implemented HHP technology in their production lines, followed by PEF technology at 20 %. HHP is mainly used for the preservation of fruit- and vegetable-derived products, egg products, dairy products, seafood, meat products and alcoholic beverages (*74*).

Pulsed electric field. PEF technology is a non-thermal method of food preservation that uses short pulses of electricity for microbial inactivation. Basically, PEF is based on the application of high-voltage pulses (20–80 kV/cm) for short periods of time (in the order of ms or μs) to a product enclosed by or flowing between two electrodes (*75*).

The mechanism of action of PEF on microorganisms is based on the disruption of the membranes by the induction of electromechanical compression that leads to the pore formation. This phenomenon is called electroporation and it can be reversible, *i.e.* the cell is only sublethally damaged and can recover, or irreversible, *i.e.* the damage cannot be repaired and the cell is inactivated (*76*, *77*). The inactivation of pathogenic and spoilage vegetative cells of bacteria and yeasts by PEF has been widely demonstrated. However, the few studies conducted on PEF inactivation of bacterial spores describe these structures as PEF-resistant and show that their inactivation is only achieved in combination with heat (*78*). For this reason, this technology is used as an alternative to pasteurisation rather than sterilisation. Therefore, PEF treatments can ensure food safety and extend the shelf life of certain foods, avoiding detrimental changes in food quality and maintaining physical, sensory and nutritional attributes compared to pasteurisation (*79*).

PEF is generally not suitable for preservation of solid food (*79*). Pasteurisation by PEF has been extensively demonstrated for various liquid food products such as fruit juices, milk and dairy products, soup, liquid egg and beverages, among others (*80*, *81*). However, this technology has several limitations in the food industry: food must be free from air bubbles and have low electrical conductivity. Moreover, PEF has proven to be useful in some processing steps such as for the extraction of compounds of interest, or in dehydration or freezing/thawing processes of solid food (*82*). Most consumers recognise and appreciate the benefits of PEF as an alternative pasteurisation method to heat, for example in apple juice. Nevertheless, there are still some consumers who have doubts about the safety of using PEF as a food preservation method (*83*), especially for traditional food such as wine.

Ionising radiation (IR). Food irradiation is a process in which food is exposed to a carefully measured amount of intense radiant energy called ionising radiation (IR), which is capable of ionising atoms or molecules by detaching electrons from them. This non-thermal technology is a preservation method with minimum effect on the quality, taste, appearance and texture of food. It assures the inactivation of pathogenic and spoilage bacteria, yeasts and moulds at the legal doses permitted in the European Union, except in their spore form (*84*, *85*).

The mechanism of bacterial inactivation in IR treatments is based on the damage to microbial DNA, either directly or indirectly (*86*). In the direct action, the IR directly hits the DNA molecules and disrupts the molecular structure. Such structural change leads to cell damage or even cell death. In the indirect action, the IR hits the water molecules, which are the major constituent of the microbial cell and other organic molecules inside the cell, and produces free radicals such as hydroxyl ion (OH˙) and hydrogen atoms (H˙). These free radicals have an unpaired electron in the structure that is very reactive and therefore reacts with DNA molecules and causes a molecular structural damage. In addition, these reactive species, also called reactive oxygen species (ROS), can damage other cellular structures, such as membranes, where alterations have been observed after IR treatment. Direct and indirect IR treatments lead to cell death of the microorganisms present in the food.

As per the General Standard for Irradiated Foods (*87*), food irradiation can be achieved using three different types of ionising radiation: (*i*) gamma rays emitted by radionuclides like cesium-137 (^137^Cs) or cobalt 60 (^60^Co), (*ii*) X-rays generated by machine sources operating at or below 5 MeV energy level and (*iii*) electron beams produced by electron accelerators at or below 10 MeV energy level (also known as e-beam).

For food irradiation, it is essential to ensure that the average absorbed radiation dose in processed food remains below 10 kGy and that none of this radiation has sufficient energy to produce radioactivity. Regarding the safety of IR, according to FAO, IAEA, WHO and the Scientific Committee on Food of the European Commission, foods irradiated with appropriate technologies and doses are safe and nutritionally adequate (*88*).

However, the use of this technology to treat food is very restricted in some countries by legal regulations. In the EU (*88*), the following foods may be treated (note that this depends on each member country): fruits and vegetables, including root vegetables; cereals, cereal flakes and rice flour; spices and condiments; fish and shellfish; fresh meat, poultry and frogs’ legs; raw milk camembert; gum arabic, casein/caseinates, egg white and blood products. It should be noted that in many cases the permitted irradiation is not aimed at microbial inactivation but at preventing or slowing down the germination of some vegetables, *e.g.* potatoes. All imported, exported, advertised, distributed, stored, manufactured and sold food items that have been irradiated, contain irradiated ingredients or have been processed from irradiated materials must bear the food irradiation logo on their labels (*87*).

IR offers several advantages over traditional heat-based or chemical food preservation methods including: treatment of packaged food, chemical and temperature independence, liquid and solid food and minimal organoleptic changes (*89*). However, IR still has some limitations for its application in some sectors of the agri-food industry, such as the legal restrictions on its use, the cost of facilities for its application, the high and costly safety measures for its safe and efficient use and the insufficient acceptance by consumers/manufacturers (*73*, *84*).

Non-thermal atmospheric plasma. NTAP is generated at room temperature and normal pressure by electrical discharge of a gas, resulting in ionisation, dissociation and excitation of its atoms and molecules. NTAP consists of numerous reactive units, such as electrons, positive and negative ions, free radicals, excited or unexcited atoms and molecules, and electromagnetic radiation (UV rays) (*90*). However, depending on the gas and the method used to generate NTAP, its constitution differs, including both ROS and reactive nitrogen species (RNS). These include ozone (O_3_), superoxide (O_2_˙̄), hydrogen peroxide (H_2_O_2_), hydroxyl (OH˙), peroxyl (ROO˙) radicals, singlet oxygen (^1^O_2_), atomic oxygen (O), nitric oxide (NO) or nitrogen dioxide (NO_2_) (*91*).

NATP has demonstrated strong antimicrobial properties against a broad group of food pathogens, such as *Listeria monocytogenes, Escherichia coli* and *Salmonella* spp. (*92*). However, the mechanism of action of the NATP components that cause microbial inactivation is not yet fully understood. Many studies have observed the oxidative damage to various structural and functional cellular components by the reactive species produced in NATP. So far, the hypothesis supporting its good antimicrobial properties against bacteria is that these reactive species act on multiple cellular targets, and the sum of all damage would lead to cell death. Many authors have observed that the components of NATP (reactive species) can cause DNA damage, cell leakage, protein modification, lipid peroxidation and morphological changes, highlighting the membrane as one of the main structures altered by this technology (*93*).

This technology has several advantages that make it one of the most studied techniques in recent years for its use in the food industry: (*i*) low application cost, (*ii*) short treatment times, (*iii*) versatility in treating food and contact surfaces, (*iv*) treatment within the packaging to avoid subsequent contamination and (*v*) environmentally friendly technique as it can use ambient air (*91*). However, there are still some limitations to this technology that hinder its application for food preservation. In this sense, it is necessary to evaluate the nutritional and sensory effects depending on the intensity of the treatment and the food, the role of individual plasma components responsible for its antimicrobial properties, the lack of short- and long-term toxicity for consumers and the development of equipment that is easy to use (*93*).

## ILLUSTRATIONS OF THE APPLICATION OF HURDLE TECHNOLOGY PRINCIPLES: COMBINATION OF ANTIMICROBIAL AND/OR ANTIOXIDANT PLANT EXTRACTS AND OTHER HURDLES

Despite advances and optimisations of non-thermal technologies, their use as the only method of food preservation sometimes requires very high treatment intensities that are not feasible in industry and/or lead to organoleptic changes in the food. For this reason, many studies are looking at combining different preservation methods (*i.e.* hurdles) to achieve synergistic effects. Successful synergism occurs when two or more hurdles are applied simultaneously and/or sequentially and the obtained bacteriostatic or bactericidal effect is greater than that achieved by the hurdles applied individually. Thus, these preservation methods would achieve the degree of inactivation necessary to ensure food safety at lower treatment intensity, with lower impact on the organoleptic and nutritional value of food.

As mentioned above, for example, the doses of essential oils and their individual constituents required for food preservation are not very high (in the order of hundreds of ppm), but even these small concentrations can lead to changes in odour and taste of the food that consumers might reject (*94*). Therefore, the development of new strategies to optimise the efficacy of essential oils and individual constituents in food preservation is focused on reducing the concentrations and thus consumer acceptance.

Among the possible methods of food preservation, one of the most promising is the combination of these technologies, whether traditional such as heat or emerging such as HHP, PEF, IR, or NATP treatments, in combination with the addition of antimicrobial compounds of natural origin. The combined use of antimicrobials has shown that their individual constituents interact effectively, and great synergistic effects have been described that can either inhibit or inactivate pathogenic or food spoilage microorganisms (*95*).

### Essential oils and existing methods (mild heat)

Numerous studies support the synergistic effect of some essential oils or their individual constituents combined with mild temperatures on the inactivation of pathogenic or food spoilage microorganisms. In fact, among the physical hurdles, heat is the most studied method in combination with essential oils and individual constituents, which show synergistic effects when applied simultaneously (*95*).

Ait-Ouazzou *et al.* (*96*) observed synergistic effects when combining heat (54–60 °C) with 0.2 μL/L *Mentha pulegium* L. or *Thymus algeriensis* L. essential oil, which reduced by 3.5 and 5.7 times, respectively, the time of inactivation of 5 log cycles of *E. coli* O157:H7 cells in apple juice. Pagan *et al.* (*97*) observed a remarkable synergism between heat and citral against *E. coli* O157:H7 at acid and neutral pH; while heat treatment at 53 °C for 15 min reduced only less than 1 log cycles of bacteria, more than 4 log cycles were inactivated with 0.1 μL/L citral. It should be noted that 0.1 μL/L citral applied without heat did not inactivate *E. coli* O157:H7 Sakai. Similar synergistic effects have been reported between essential oils and heat against non-pathogenic *Escherichia coli* (*98*), *Listeria monocytogenes* (*99*) or *Cronobacter sakazakii* (*100*). A recent study has demonstrated that the combination of heat and essential oils can inactivate even heat-resistant microbial variants in coconut water; the addition of 200 μL/L carvacrol increased the thermal reduction of heat-resistant variants of *E. coli* O157:H7 in coconut water at 57 °C from less than 2 log units to more than 5 log units (*101*).

In addition, the synergism between heat and essential oils or individual constituents has been demonstrated in sessile cells, which are usually highly resistant to common disinfectants due to the protection by the biofilm-forming polysaccharides (*102*). This study showed the inactivation of more than 5 log cycles of sessile cells that are part of mature biofilms of *Staphylococcus aureus* SC-01, *Listeria monocytogenes* EGD-e or *Escherichia coli* MG1655, after treatments with either carvacrol or citral (1000 μL/L) at moderate temperatures (45 °C). Some studies have shown large synergistic effects that allow the temperatures and the concentrations of essential oils or individual constituents to be reduced to intensities and dosages that are sensorially acceptable to consumers in fruit juices and vegetables soup (*94*, *103*).

In order to improve the chemical stability of essential oils and reduce the required doses, several studies have investigated the use of these antimicrobials in emulsified or encapsulated form in combination with heat. While the use of citral emulsified with Tween^®^ 80 reduced the synergistic effect with heat compared to the use of citral in its free form (*97*), orange essential oil in its emulsified form with chitosan showed an increased bactericidal effect in apple juice (*104*). As for encapsulation, Merino *et al.* (*105*) reported that encapsulation with zein reduced the bactericidal effect of thyme essential oil. However, when these encapsulations were used in combination with heat at 53 °C, synergistic effects against *E. coli* and *L. monocytogenes* were observed to an even greater extent than with the essential oil in free form.

Cell membrane disruption and loss of cell membrane potential are considered to be the main causes of bacterial inactivation by heat and individual constituents (*106*). However, it is very likely that the basis of the strong synergy between heat and essential oils or individual constituents is caused by multiple damages and/or alterations of other cellular structures or functions targeted by both preservation methods.

### Essential oils and emerging methods

Emerging technologies for food preservation are proposed as an alternative to heat treatments because they have minimal or little impact on food quality. However, these technologies alone are not always able to ensure food safety and extended shelf-life. For this reason, many authors are pursuing the development of strategies based on the combination of these physical methods with natural antimicrobials, such as essential oils or their individual constituents (*107*).

HHP was combined with essential oil, which led to interesting results. According to Espina *et al.* (*108*), the combination of HHP treatments (175–400 MPa for 20 min) with 200 μL/L of each essential oil (*Citrus sinensis* L., *Citrus reticulata* L., *Thymus algeriensis* L. and *Rosmarinus officinalis* L.) or their individual constituents ((+)-limonene and carvacrol) were able to inactivate about 4–5 log cycles of the initial cell populations of *Escherichia coli* O157:H7 and *Listeria monocytogenes* in orange and apple juices. Another study showed that the addition of nanoemulsified *Mentha piperita* essential oil improved the lethality of HHP treatments against *E. coli* O157:H7 in tropical fruit juices (*109*). Synergistic effects were also observed with HHP treatments (200 MPa) in combination with cauliflower or mandarin infusion against *S. typhimurium* (*110*).

It is likely that the observed synergism between HHP and essential oils is based on the damage caused to one of the main targets of both preservation methods: the cell envelopes. The accumulation of damage in this structure could be the reason for the increased antimicrobial efficacy when both technologies are used in combination (*109*). Other studies indicated that HHP can also alter membrane structure, leading to a reduction in the microbial resistance to natural antimicrobials (*111*).

However, not all combinations of HHP and natural antimicrobials always show synergism. For example, according to Bleoanca *et al.* (*112*), a combined treatment at 200–300 MPa with thyme extract did not improve the antimicrobial effect. Thus, a synergistic, additive or antagonistic effect can be observed when HHP is combined with antimicrobial compounds, depending on the antimicrobial compound, its mode of action and dosage (*113*).

Regarding PEF treatments, several studies reported a synergistic effect when this non-thermal technology is combined with essential oils or their individual constituents. According to Wang *et al.* (*114*), a synergistic effect was observed between PEF and carvacrol against *S. aureus* when individual constituents were added to the recovery agar medium. Enhanced antimicrobial activity of PEF in combination with citral (0.2 µL/mL) was described against *E. coli* (30 kV/cm) and with mandarin and cauliflower infusion (5 %) against *S.* Typhimurium (20 kV/cm) (*115*). It is likely that the electroporation triggered by PEF treatment enhances the effect of essential oils or individual constituents on the cell membrane and even allows the entry of the natural antimicrobials into the cytoplasm, increasing its antimicrobial activity (*116*).

However, some studies have reported that the combination of PEF with essential oils or individual constituents does not always have a synergistic lethal effect. For example, Somolinos *et al.* (*117*) observed that the lethal effect of PEF treatment to inactivate *E. coli* was the same in the presence or absence of citral (200 μL/L). In this context, some studies suggest that the synergistic effect of PEF and essential oils or individual constituents depends on the way the antimicrobial agents are used (*e.g.* emulsified) and the design of the combined treatment (simultaneous or sequential treatment). Several studies suggest that the addition of essential oils and individual constituents in emulsified form may increase the synergistic effect of combined treatment with PEF (*109*, *117*). Furthermore, Clemente *et al.* (*118*) found that the simultaneous application of PEF and an essential oil did not have any synergistic effect on microbial inactivation, whereas this was the case when the essential oil was added after PEF treatment.

Nevertheless, the magnitude of the synergistic effect when combining essential oils or individual constituents with PEF is usually smaller than with heat (*109*). Under the same experimental conditions, citral in combination with heat produced a synergistic effect more than three times greater than that observed with PEF against *E. coli* O157:H7 (*97*).

As for the IR treatments with essential oil and individual constituents, synergistic effects were observed with the combined use of both hurdles for microbial control of foodborne pathogenic bacteria (*Listeria monocytogenes*, *Salmonella* spp., *Staphylococcus aureus*, *Bacillus cereus* and *Vibrio* spp.) contaminating flour, fish, maize and rice (*107*).

The combination of cinnamon essential oil (3 %) encapsulated in alginate with gamma IR (1.5 kGy) has shown synergistic effects in inhibiting a cocktail of resistant *Escherichia coli* O157:H7 strains in dry fermented sausages during ripening and shelf life after vacuum packaging (*119*). Begum *et al.* (*120*) also reported an increase in the antimicrobial effect of gamma and X-ray irradiation (up to 1500 Gy) against *Escherichia coli*, *Salmonella* Typhimurium and *Listeria monocytogenes* in rice when combined with oregano/thyme essential oil. Moreover, the combination of IR with essential oil has also been studied against moulds with promising results. Shankar *et al.* (*121*) observed the potential of a mixture of oregano and thyme essential oil to increase the radiosensitisation not only of bacteria, *Bacillus cereus* and *Paenibacillus amylolyticus*, but also of moulds, *Aspergillus niger*, during treatment with gamma rays or X-ray irradiation.

In recent years, the antimicrobial properties of essential oils in edible food coatings have been further explored, as well as their combination with other preservation technologies such as IR. According to Abdeldaiem *et al.* (*122*), the combined effect of gamma IR (1 kGy) and coatings containing mass fraction of 0.5 % rosemary (*Rosmarinus officinalis*) essential oil reduced the number of Enterobacteriaceae, *Staphylococcus aureus*, *Bacillus cereus*, *Vibrio* spp. and *Salmonella* spp. Hossain *et al.* (*123*) also observed a synergistic effect against moulds when combining both preservation methods; chitosan-based nanocomposite films loaded with thyme and oregano essential oils showed significantly higher antifugal activity against *Aspergillus niger*, *Aspergillus flavus*, *Aspergillus parasiticus* and *Penicillium chrysogenum* in rice at a dose of 750 Gy compared to treatment with the bioactive film or IR alone.

On one hand, according to Ayari *et al.* (*124*), IR enhances the microbial inactivation of the essential oil by altering the integrity of the cell membrane and facilitating the entry of antimicrobial compounds, thereby increasing cell damage. On the other hand, the mechanisms of IR and essential oil or individual constituents include an increase in the generation of intracellular ROS in bacterial cells and consequently increased cell damage (*120*). In this sense, it is likely that the generation of intracellular ROS by both preservation methods leads to a synergistic effect that enhances antimicrobial activity in combined processes.

As for NATP with essential oil, there are not many publications that have investigated their combination against foodborne pathogenic bacteria in food. Nevertheless, some authors have observed an important synergistic effect of NATP in combination with natural antimicrobials. According to Matan *et al.* (*125*), the antibacterial activity of clove oil, sweet basil oil and lime oil was enhanced by NATP (20–40 W for 10 min) to effectively control the growth of *Escherichia coli*, *Salmonella* Typhimurium and *Staphylococcus aureus* on chicken egg. The best results were observed with clove oil and NATP at 40 W, which completely inhibited all bacteria tested in the study. Moreover, the combination of essential oil and NATP was also tested to control biofilm formation of foodborne pathogenic bacteria such as *E. coli* and *S. aureus* (*125*). Getnet *et al.* (*126*) demonstrated that the deposition of carvacrol thin film on stainless steel by NATP completely inhibited *E. coli* biofilm formation and reduced *S. aureus* adhesion by six orders of magnitude, which was a slight improvement over the use of carvacrol alone. However, it should be noted that this synergistic effect was not due to an interaction of the mechanisms of action of NATP and essential oil on the bacteria, but to an enhancement of the deposition of carvacrol, thus preventing biofilm adhesion and growth (*126*).

### Plant extracts and modified atmosphere or vacuum packaging

In vacuum packaging the air is removed from the package before it is sealed. This procedure reduces the atmospheric oxygen in the packaged foods, thereby limiting the growth of aerobic microorganisms and oxidation of foods. However, soft or spongy foods such as salads or soft-crust bread are too delicate to withstand vacuum packaging. Another drawback of vacuum packaging of foods such as raw beef results from the purple colour of deoxymyoglobin, while consumers associate the bright red colour of raw beef (due to oxymyoglobin) with its freshness.

Unlike vacuum packaging, modified atmosphere packaging offers the possibility to choose the initial gas composition of the inner atmosphere of packaged foods. For example, high-oxygen modified atmosphere packaging (HOMAP) allows the preservation of the red colour of raw beef for a longer time. The addition of carbon dioxide at volume fractions higher than 20 % in modified atmosphere is another way to extend the shelf life of refrigerated foods susceptible to microbial deterioration by taking advantage of their bacteriostatic and fungistatic properties above this volume fraction. However, too high carbon dioxide volume fractions can lead to mild acidification of foods resulting from partial carbon dioxide solubilisation in food matrices (*127*). Nitrogen is an inert gas with limited solubility in water and fat, and it is usually used to replace oxygen or in addition to carbon dioxide to prevent package collapse due to carbon dioxide partial solubilisation in foods.

The possibility to further extend the shelf life of perishable foods packaged under vacuum or modified atmosphere by adding natural antimicrobial molecules, including plant extracts, has already been pointed out by Mastromatteo *et al.* (*128*) in their review. However, most studies investigating the potential of modified atmosphere packaging in combination with plant extracts have been published in the last decade (Table 1 (*129*-*143*)). The potential of such combinations has been tested mainly for the preservation of high value-added foods prone to rapid spoilage such as fish, seafood, poultry and meat. Increasing interest in the use of the antimicrobial and/or antioxidant activity of plant extracts has been stimulated by concerns about the toxicity of food preservatives and antioxidants, such as nitrites or sulfites. The plant extracts used can be an essential oil (usually produced by steam distillation and containing only volatile constituents) or extracts prepared with solvents of different polarity (water, ethanol, supercritical CO_2_, *etc*). Most plant extracts contain phenolics that give them antioxidant activity, which is particularly interesting for prolonging the shelf life of red meat stored in HOMAP (*129*-*137*) or fish and seafood stored in MAP containing oxygen (*140*). Moreover, many essential oils (*144*) and phenolic-rich edible plant extracts (*145*) exert antimicrobial activity against undesirable microorganisms that can extend the shelf life of perishable foods. While plant extracts almost always exhibit antioxidant activity resulting from their phenolic constituents, not all plant extracts that have antimicrobial activity *in vitro* in microbiological media exert this antimicrobial activity in food (*130*, *132*). This is likely due to the interactions of the antimicrobial constituents of plant extracts with some food components (complexation of phenolics by some food proteins, interaction of hydrophobic essential oil with dispersed fat). These interactions would limit the quantity of these molecules accumulating on the surface of the target microorganisms, thereby inhibiting their growth or leading to cell lysis by different mechanisms of action (*146*-*148*). The sometimes reported lack of the effect of plant extracts on total viable count during storage of modified atmosphere packaged foods could also be due to the fact that total viable count refers to different microorganisms in the complex microbial ecosystem of such foods. Therefore, plant extracts should have a broad spectrum of antimicrobial activity to significantly reduce the total viable counts during refrigerated storage of such foods. An analysis of the literature shows that while plant phenolics are generally effective antioxidants in food matrices, plant phenolics that inhibit the growth of certain microorganisms *in vitro* in microbiological media are not always effective in food matrices, which have a far more complex composition and microstructure. Nevertheless, since not only microbial spoilage of perishable foods but also their oxidation alters their organoleptic quality and limits their shelf life, the addition of plant extracts generally results in a further extension of the shelf life of foods packaged under modified atmosphere. However, a frequently reported limit to their use is their effect on sensory properties (*137*, *138*). Their taste or odour can be excessive at concentrations that effectively inhibit unwanted microorganisms in foods. In this context, the use of mixtures of antimicrobial plant extracts selected for their synergistic antimicrobial activity has been proposed to reduce the amount of individual plant extracts. In line with hurdle technology principles, several authors have considered additional hurdles besides the combination of refrigeration, modified atmosphere packaging and the addition of antimicrobial and antioxidant plant extracts. Olatunde *et al.* (*140*) took advantage of an antioxidant plant extract to limit the defects caused by oxidation of fish during the 5-minute cold plasma treatment. Frangos *et al.* (*142*) combined the addition of oregano essential oil with the reduction of water activity by salting trout fillets. Mahdavi and Ariaii (*143*) combined grape pomace extracts with nisin to extend the shelf life of fish sausage. Interestingly, Kurek *et al.* (*149*, *150*) used the volatility of the antimicrobial essential oil constituents to control their release in the vapour phase, which opens the prospect of placing an essential oil reservoir in modified atmosphere packaging system. This reservoir can be an induction layer with essential oils on the inside of food packaging films.

**Table 1:** Examples of the effects of the addition of plant extracts/phenols on the shelf life/properties of modified atmosphere (MAP) or vacuum packaged foods

Food type	Modified atmosphere composition *φ*/%	Plant extract/ phenol	Effect on food shelf life/properties	Reference
Ground beef	O_2_ 80, CO_2_ 20 high oxygen MAP	Tannic acid	Treatment with *w*(tannic acid)=200 mg/kg resulted in a lower number of thiobarbituric acid-reactive substances and psychrophilic bacterial count and had the highest likeliness score for colour after 15 days of storage at 4 °C	(*129*)
Ground beef patty	O_2_ 70, CO_2_ 20, and N_2_ high oxygen MAP 10	Prune flesh, pomegranate peel, green tea leaves, grape seed extracts, and Gaillac red wine powder	Addition of *w*(plant extract)=1 % did not affect microbial counts (total viable counts, psychrotrophic aerobic bacteria) during 12 days of storage at 4 °C No increase in thiobarbituric acid-reactive substances was observed for 12 days in patties with *w*(green tea)=1 %, pomegranate peel or grape seed extract and Gaillac red wine powder	(*130*)
Bovine meat patty	O_2_ 80, CO_2_ high oxygen MAP 20	Sugarcane bagasse, orange peel and tomato pomace	Sugarcane bagasse, orange peel and tomato pomace reduced oxidative deterioration of colour, lipids and proteins and delayed the growth of microorganisms for 12 days at 4 °C	(*131*)
Ground bovine meat (*w*(patty)=85 % and *w*(pork fat)=15 %)	O_2_ 80, CO_2_ high oxygen MAP 20	Grape seed extract	The addition of *w*(grape seed extract)=0.75 g/kg improved the colour stability, inhibited the lipid and myoglobin oxidation of raw patties but did not affect total viable counts for 10 days of storage at 4 °C	(*132*)
Pork loin	O_2_ 80, CO_2_ high oxygen MAP 20	*w*(gallic acid)=0.2 % with *w*(nisin)=0.2 % in a chitosan coating	Lowest thiobarbituric acid-reactive substances values and total viable count after 20 days of cold storage when gallic acid and nisin were incorporated together into chitosan films	(*133*)
Raw pork patty	O_2_ 80 and CO_2_ high oxygen MAP 20	Oak wood extract	Addition of up to *w*(extract)=1 % inhibited the growth of enterobacteria, lipid oxidation (including a decrease of the volatile compounds resulting from their oxidation reactions) during 12 days of storage at 4 °C Modifications of organoleptic properties after the addition of oak wood were well accepted	(*134*)
Pork burger	O_2_ 80, CO_2_ high oxygen MAP 20	Pitanga leaf extract	Untargeted metabolomics approach was used to study the effect of adding *w*(pitanga leaf extract)=250 mg/kg to pork burgers during 18 days of storage at 2 °C; its lipid antioxidant effect was confirmed	(*135*)
Lamb cutlet	O_2_ 50, CO_2_ 30 and N_2_ high oxygen MAP 20	North-African essential oils from *Pituranthos chloranthus* and *Teucrium ramosissimum*	Spraying the surface of lamb cutlets with 1 % essential oil solutions in *V*(ethanol):*V*(water)=70:30 resulted in a decrease of total viable counts, *Enterobacteriaceae*, lactic acid bacteria and thiobarbituric acid-reactive substances during 12 days of storage at 4 °C	(*136*)
Poultry fillet	N_2_ 70, CO_2_ MAP 30	Oregano essential oil and grapefruit seed extract	0.5 % oregano essential oil with 0.1 % grapefruit seed extract effectively inhibited *Listeria monocytogenes* and to a lesser extent *Salmonella* Typhimurium, but negatively affected sensory properties	(*137*)
Broiler chicken cut	CO_2_ 65, N_2_ MAP 35	Ethanol extracts of Finnish sea buckthorn berries or lingonberries, supercritical CO_2_ˉ extracts from a commercial blend or oregano leaves	Fresh brined skinless broiler chicken breast cuts in marinade had a final *w*=0.2 % of each extract. Commercial blend and oregano extracts inhibited the growth of lactic acid bacteria unlike sea buckthorn and lingonberry extracts. Nevertheless, the dose of the commercial blend and oregano leaf extract should be adjusted because of their strong taste	(*138*)
Cooked sausage made from pork meat, emmer wheat, almond and hazelnut	Vacuum-packed	Commercial mix of pomegranate and *Citrus* spp. extracts	Addition of *w*(mix)=1 % resulted in: (*i*) a significant decrease in total viable and psychrophilic microbial counts and lactobacilli during 60 days of cold storage and (*ii*) a 16-day extension of shelf life estimated from sensory analysis	(*139*)
Asian sea bass slice	CO_2_ 60, Ar_2_ 30, O_2_ MAP 10	Ethanol coconut husk extract encapsulated in liposomes	A synergistic effect of the addition of 400 ppm ethanol coconut husk extract encapsulated in liposomes with a cold plasma treatment for 5 min before packaging resulted in the extension of shelf-life for more than 18 days at 4 °C	(*140*)
Mediterranean octopus (*Octopus vulgaris*)	Vacuum packaging	Oregano essential oil	Treatment with *w*(oregano essential oil)=0.2 and 0.4 % resulted in: (*i*) a significant reduction of total aerobic plate count, *Pseudomonas* spp., H_2_S-producing bacteria, lactic acid bacteria and *Enterobacteriaceae* and (*ii*) extension of shelf life for 8 and 17 days	(*141*)
Trout fillet	Vacuum packaging	Oregano essential oil with salt	Shelf life was extended from 14 to 16-17 days after the addition of *w*(oregano essential oil)=0.2 %	(*142*)
Fish sausage produced from silver carp (*Hypophthalmichthys molitrix*)	CO_2_ 70, N_2_ MAP 30	Grape pomace extract with nisin	Decrease in total viable count, psychotropic bacterial count and *Clostridium botulinum* during 42 days of storage at 4 °C	(*143*)

### Essential oils and biopreservation

The antimicrobial activity of essential oils occurs in several ways depending on the wide variety of components that they contain. The main target in all microbial cells is the cytoplasmic membrane (*151*), from which permeability is influenced by its composition and the hydrophobicity of the compounds that pass through it. Thus, due to the hydrophobic nature of essential oils, the activity of these compounds triggers structural and functional damage to the cytoplasmic membrane of bacterial cells and changes its structure and fluidity (*151*). The consequences are dissipation of proton motive force with regard to the reduction of the ATP pool, internal pH disorder, electrical potential perturbation and loss of metabolites and ions, such as potassium and phosphate, ultimately culminating in cell death (*151*).

The antibacterial mechanisms of essential oils depend not only on their respective structures, chemical constituents and functional groups but also on their synergistic interactions with other preservative molecules or bioprotective cultures. Moreover, the mode of action of essential oils against Gram-positive and Gram-negative bacteria differs, which is related to the structure and composition of the outer membrane of cell walls (*152*).

The effect of essential oils in food as biopreservatives depends on various associated factors, such as the form of application, the concentration applied, the pathway of action, the storage conditions and the methods of application, such as spraying, immersion and embedding in lactose capsules (*42*).

Food biopreservation is a method of food preservation that uses various natural ingredients and metabolites with antimicrobial activity. Moreover, through the application of antimicrobial substances produced by beneficial microorganisms released during a targeted fermentation, biopreservation is a method that both has a preservative effect on the food and can provide the food product with functional and health-promoting properties. This dual beneficial effect can be achieved by incorporating ingredients with proven preservative and functional effects into the food product, which can act as separate hurdles for achieving biopreservation and agents that make the developed food product a functional food. A good example of this is the development of successful biopreservation strategies for different food products by the application of essential oils from various plant species and probiotic bacteria. Our team has studied the synergistic effect of selected probiotic lactic acid bacteria and essential oils with high antimicrobial activity against pathogenic and spoilage microorganisms for the biopreservation of chocolate mousse emulsion and egg-free mayonnaise emulsion (*153*-*155*).

Three methods of biological preservation of mayonnaise or chocolate mousse were used: (*i*) with probiotic bacteria only, by incorporation of free and encapsulated cells into the food emulsion, (*ii*) with essential oil only and (*iii*) with a combination of probiotic bacteria and essential oil. The obtained chocolate mousse variants had preserved organoleptic properties and microbiological safety. Free or encapsulated probiotic *Lactobacillus plantarum* D2 cells applied alone or in combination with lemon or grapefruit essential oil provided biopreservation of the chocolate mousse emulsions, maintaining a high concentration of viable cells (10^6^–10^7^ CFU/g) during 20-days storage under refrigerated conditions (*153*). The mayonnaise variants preserved with *L. plantarum* LBRZ12 maintained a high concentration of viable cells of the probiotic strain during storage (*154*). In the development of the chocolate mousse variants, the combined application of free or encapsulated probiotic LAB and lemon or grapefruit essential oil resulted in better biopreservation than the use of probiotic LAB or essential oil alone, thus suggesting a synergistic effect between the two biopreservative agents. Moreover, the obtained chocolate mousse emulsions could be classified as functional foods and the chocolate mousse food matrix can be successfully used as a vehicle for delivery of probiotic LAB to a wide range of food consumers (*153*). A similar trend was observed in the development of the mayonnaise variants, thus confirming the synergistic effect of probiotic bacteria and essential oils as two hurdles used for the development of a successful biopreservation strategy for the two food emulsion types (*154*).

Since the antimicrobial activity of a given agent determined *in vitro* is usually higher than the antimicrobial activity of the same agent determined *in situ*, it was of great importance to examine the changes in the dynamics of a mixed population of a pathogen and a probiotic strain by a microbial challenge test. Two parallel experiments were conducted: *in vitro* examination of the changes in the population dynamics in complex nutrient medium (MRS broth) and *in situ* tests of the changes in the population dynamics in chocolate mousse food matrix at two temperatures (4±2) and (20±2) °C. The conducted study demonstrated that the use of free or encapsulated probiotic *Lactobacillus helveticus* 2/20 cells for biopreservation of chocolate mousse is a promising hurdle to reduce pathogen survival (of *E. coli* ATCC 25922 and *S. aureus* ATCC 25923). In addition, the results of the *in situ* and *in vitro* studies showed that the inhibition in MRS broth was similar to that in the chocolate mousse stored at (20±2) °C. Nevertheless, the effect of pathogen inhibition at (4±2) °C was different between the *in vitro* and *in situ* studies. As expected, the effect of pathogen inhibition was weaker in the food matrix than in the nutrient medium. This conﬁrms the fact that an *in situ* evaluation is imperative before conﬁrming the biopreservation potential of any bioprotector (*155*).

## COOPERATION AND PERSPECTIVES

**Fig. 2:**
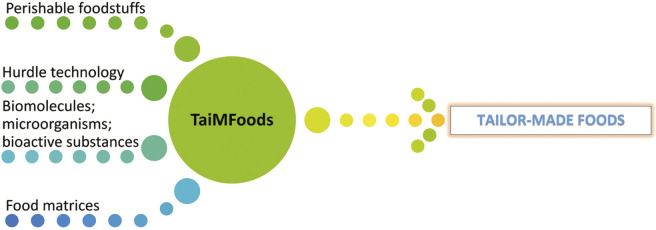
Basic tasks of TaiMFoods project

To study the minimal processes for the production of tailor-made foods, a project was developed and financed within the framework of the national programme European Scientific Networks, funded by the Ministry of Education and Science of the Republic of Bulgaria. The project proposed a concept called TaiMFoods (Fig. 2) to investigate different aspects of the production and preservation of tailor-made foods. This concept is based on the scientific expertise of individual project partners and enables the building of a multidisciplinary team for the development of new types of food through the application of established and new technologies. The development and implementation of the TaiMFoods project is related to the defined strategic directions for scientific research in the Republic of Bulgaria 2017–2030. An important element of the strategy is the development of new technologies in the field of food technology and biotechnology with the aim of improving the quality of life through the introduction of sustainable and healthy food production processes. The strategy promotes the formation of consortia of scientific, educational and development institutions to conduct high-quality scientific research in strategic areas. The implementation of the TaiMFoods project is precisely such a strategic partnership.

The building of the consortium aims to develop a constantly evolving and sustainable research platform in the field of food production with programmable properties. Taking into account the different directions of work, the scientific objectives of the consortium focus on carrying out research in the following directions: (*i*) mechanism of biological activity of biomolecules and microorganisms in the development of foods with programmable properties (including bioactive substances derived from waste products of the food industry), (*ii*) processing methods to increase food safety and shelf life of tailor-made foods by applying biopreservation, hurdle technologies and minimal food processing and (*iii*) methods for analysis of the distribution and activity of biomolecules and microorganisms in food matrices.

## CONCLUSIONS

Food systems with programmable properties (tailor-made foods) are systems that have not only nutritional but also certain functional properties related to human health. Their production requires an integrated approach to the evaluation of nutritional and functional properties, the creation of databases of the various ingredients and their influence on food functionality. Therefore, it is important to form teams of different specialists working on targeted projects for the successful development of tailor-made foods. The development of tailor-made foods that are minimally processed to preserve both the organoleptic quality and the bioactive constituents of the food, as well as to incorporate the ingredients, such as probiotic microorganisms or some plant extracts that have a positive effect on both the shelf life of the food and its content of health-promoting components is promising. The rational development of such tailor-made foods requires the identification of the mechanisms of action of antimicrobial molecules and/or classical and emerging food preservation methods. The principles of hurdle technology for the preservation of perishable foods make it necessary to combine preservation methods and/or antimicrobial agents with different molecular mechanisms of action. Indeed, it is suggested that hurdles with different mechanisms of action are more likely to act synergistically. The combination of biological hurdles such as probiotic cultures and essential oils is of particular importance for the development of specialised tailor-made foods and products. Appropriate combinations of these two hurdles not only provide a biopreservative effect, but create products with potential health benefits for the human body.
